# Prooxidant Properties of p66shc Are Mediated by Mitochondria in Human Cells

**DOI:** 10.1371/journal.pone.0086521

**Published:** 2014-03-11

**Authors:** Evgeny R. Galimov, Boris V. Chernyak, Alena S. Sidorenko, Alesya V. Tereshkova, Peter M. Chumakov

**Affiliations:** 1 Belozersky Institute of Physical and Chemical Biology, Moscow State University, Moscow, Russia; 2 Engelhardt Institute of Molecular Biology, Russian Academy of Sciences, Moscow, Russia; 3 Lerner Research Institute, Cleveland Clinic Foundation, Cleveland, Ohio, United States of America; 4 Novosibirsk State University, Novosibirsk, Russia; University of Pittsburgh School of Medicine, United States of America

## Abstract

p66shc is a protein product of an mRNA isoform of *SHC1* gene that has a pro-oxidant and pro-apoptotic activity and is implicated in the aging process. Mitochondria were suggested as a major source of the p66shc-mediated production of reactive oxygen species (ROS), although the underlying mechanisms are poorly understood. We studied effects of p66shc on oxidative stress induced by hydrogen peroxide or by serum deprivation in human colon carcinoma cell line RKO and in diploid human dermal fibroblasts (HDFs). An shRNA-mediated knockdown of p66shc suppressed and an overexpression of a recombinant p66shc stimulated the production of ROS in the both models. This effect was not detected in the mitochondrial DNA-depleted ρ0-RKO cells that do not have the mitochondrial electron transport chain (ETC). The p66shc-dependent accumulation of mitochondrial ROS was detected with HyPer-mito, a mitochondria-targeted fluorescent protein sensor for hydrogen peroxide. The fragmentation of mitochondria induced by mitochondrial ROS was significantly reduced in the p66shc deficient RKO cells. Mitochondria-targeted antioxidants SkQ1 and SkQR1 also decreased the oxidative stress induced by hydrogen peroxide or by serum deprivation. Together the data indicate that the p66shc-dependant ROS production during oxidative stress has mitochondrial origin in human normal and cancer cells.

## Introduction

Mitochondria are multifunctional organelles that play a key role in energy production, apoptosis, thermogenesis, calcium signaling and metabolism of reactive oxygen species (ROS) [1]. Mitochondrial dysfunction impairs the activity of cells, tissues and organs, and participates in a remarkably wide range of pathologies commonly associated with old age and aging [2]–[4]. Manifestations of the aging process include among others a progressive loss of cells due to apoptosis induced by oxidative damages. Excessive production of ROS in dysfunctional mitochondria is believed to be the major cause of damage that contributes to diseases [5]–[7].

Initial ROS are generated mainly at complexes I and III of the mitochondrial respiratory chain in the form of superoxide, which is then converted to hydrogen peroxide by superoxide dismutase SOD2 [6], [7]. Mitochondrial genome is extremely susceptible to damaging effects of ROS produced in mitochondria as it has a limited DNA repair capacity [8]. Impaired mitochondria with mutated mtDNA tend to accumulate in aging tissues further contributing to the aging process [9]–[11].

Generation of excessive ROS seems not to be solely a consequence of mitochondrial dysfunction. Hydrogen peroxide serves as a signaling molecule and participates in numerous physiological responses such as antimicrobial defense, inflammation, cell migration cell proliferation, angiogenesis, regulation of gene expression etc. [12]. NADPH-dependent oxidases (NOXs) coupled to many membrane-bound receptors are believed to be a major source of physiologically produced ROS [13]. Activation of NOXs produces a transient increase in intracellular ROS that are removed by the thioredoxin reductase/peroxiredoxin system [14]. Recently mitochondria were also suggested to play a role in the physiological ROS signaling [15]–[17]. Export of mitochondrial ROS to cytosol could be physiologically controlled by regulation of electron transfer at different steps of the respiratory chain [15]. However, particular mechanisms of physiological ROS generation in mitochondria are poorly understood and require additional studies.

The use of ROS in physiological signaling may be associated with a trade off in protection against oxidative damage, which contributes to an acceleration of aging and a shorter lifespan. A candidate putative mediator of the mechanism is p66Shc, a product of an alternatively spliced transcript from the *SHC1* gene. While two major protein isoforms encoded by the gene, p54Shc and p46Shc, act as adapter proteins linking activated receptor tyrosine kinases to Ras in the mitogen-activated protein kinase pathway, a fraction of p66Shc isoform is targeted to the mitochondrial matrix where it is implicated in the regulated increase in intracellular ROS levels. Functions of the p66Shc isoform has attracted particular attention because its homozygous knockout in mice increases resistance to oxidative stress induced by paraquat and increases the life span by 30% [18]. The ablation of p66shc enhances cellular resistance to apoptosis induced by hydrogen peroxide, ultraviolet light [18] and staurosporine [19], all associated with the excessive production of ROS The data indicate that p66shc participates in mechanisms of oxidative stress response that may contribute to the aging process.

Further studies with the p66-/- MEFs model have identified potential role of p66Shc in ROS homeostasis. No effects of p66Shc were detected to any ROS scavenging systems [20]–[22]. In mitochondria p66shc associates with mitochondrial HSP70 protein [19], [23], and oxidative stress induces release of p66shc from the complex and the interaction with cytochrome *c.* Acting as an oxidoreductase p66shc transfers electrons from cytochrome *c* to molecular oxygen leading to generation of ROS. The produced ROS initiate mitochondrial permeability transition, cytochrome *c* release to cytosol and the induction of apoptosis [19], [22], [23]. The mitochondrial translocation of p66shc is also induced by oxidative stress, through phosphorylation by PKCβ or JNK [19], [24], [25].

Hypothetically, the pro-oxidant activity of p66shc might play a role in physiological signaling. The increase in intracellular ROS mediated by p66shc stimulates the Akt-directed phosphorylation and inactivation of fork-head transcription factors Foxo1 and Foxo3a. The inactivation of Foxo can further increase intracellular ROS as these transcription factors stimulate ROS scavenging and increase resistance to oxidative stress [26]. Similarly,, in normal adipocytes insulin treatment induces translocation of p66shc to mitochondria and stimulation of mitochondrial ROS release leading to a transient inactivation of PTEN, upregulation of Akt, which phosphorylates and inactivates of Foxo transcription factors [27]. Besides, p66shc can suppress Foxo3a through an Akt-independent mechanism [28]. The inactivation of Foxos contributes to oxidative stress through attenuated expression of the ROS scavenging enzymes catalase, glutathione-peroxidase 1 and MnSOD [29]–[31].

There is a cross-talk between p66shc and the membrane-bound NOXs [32] that significantly contribute to the stimulation of ROS production [13]. Along with other *SHC1* gene isoforms p66shc is involved in activation of Ras. Phosphorylation of p66shc at Ser36 promotes a release of SOS1 protein from its complex with Grb2 [33], an activation of Rac1 GTPase and stimulation of ROS production by NOXs [32], [34]. In turn, the activated Rac1 blocks ubiquitination and degradation of p66shc [32] leading to its accumulation and augmentation of the pro-oxidant effects in mitochondria.

Finally, p66shc is required for a pro-oxidant anticancer activity of diallyl trisulfide (DATS), an organosulfur compound contained in garlic [35]. DATS induces a rapid degradation of an iron storage protein ferritin leading to a release of a reactive iron and a production of ROS in the Fenton reaction [36].

The above evidences indicate that despite the numerous hypothetical links the exact mechanisms by which p66shc affects redox homeostasis and contributes to oxidative stress, as well as the origin and the source of ROS are still obscure. Moreover, the p66shc-dependant mitochondrial ROS accumulation so far has been demonstrated only in mouse models, which makes an immediate projection of the data to human models poorly justifiable. In the present study we analyzed contribution of mitochondria to the p66shc-induced ROS production in two human cell models treated with either hydrogen peroxide, or exposed to a serum deprivation.

## Materials and Methods

### Cell lines

Human colon carcinoma cell line RKO was from ATCC (CRL-2577). Human dermal fibroblasts immortalized by lentivirally transduced hTERT were obtained from Dr Y. Yegorov (Moscow) [37]. They passed through ∼120 doublings. The cells were maintained in DMEM supplemented with 10% fetal bovine serum. Mitochondria depleted (ρ0) RKO cells [38] were essentially free of mitochondiral DNA as confirmed by PCR detecting less than 0.1 copy of mtDNA per cell. The cells were cultured as described [39].

### Lentiviral vector constructs

Lentiviral constructs for expression of human p66shc isoform were made by ligation of a cDNA fragment containing full-length coding region of p66shc into the lentiviral vector pLCMV [40] using XbaI and BglII sites.

A selective suppression of p66shc expression was achieved by RNA interference through the introduction of lentiviral constructs expressing specific short hairpin oligonucleotides governed by the H1 RNA promoter. The following pairs of oligonucleotides were introduced by ligation to BamHI and EcoRI sites in the pLSLP vector [41]:

shRNAs targeting nucleotide residues 27–46 of the protein coding region of variant 1 transcript from the human *SHC1* gene (NM_183001):

shRNAp66 sense:

5′ gatccgtGTACAATCCACTCCGGAATGtcaagagCATTCCGGAGTGGATTGTACtttttg;

shRNAp66 antisense:

5′ aattcaaaaaGTACAATCCACTCCGGAATGctcttgaCATTCCGGAGTGGATTGTACacg;

Control shRNA transcript representing a random sequence:

shCtrl-sense:

5′ gatccgtTTCTCCGAACGTGTCACGTtcaagagACGTGACACGTTCGGAGAAtttttg;

shCtrl-antisense:

5′ aattcaaaaaTTCTCCGAACGTGTCACGTctcttgaACGTGACACGTTCGGAGAAacg.

### Cell transfection and selection

Lentiviral constructs were introduced by transient transfection of 293T cells along with lentiviral packaging plasmids pCMV-deltaR8.2 and pCMV-VSV-G using Lipofectamin LTX reagent (Invitrogen) as previously described [42]. Viral particles were harvested starting 24 hours after the transfection and used for infection of target cells with the addition of 5 µg/ml polybrene (Sigma). Transduced cells were selected with the regular growth medium containing 1 µg/ml puromycin (Sigma).

### Detection of reactive oxygen species

Twenty four hours before experiment, cells were seeded into a six-well plate (80,000 cells per well). Cells were exposed to 200–1200 µmol hydrogen peroxide for 3 hours, or placed in serum-free medium for 24 hours. Subsequently, cells were incubated with the fluorescent dye DCF-DA (Sigma, 10 µmol) for 30 min, detached with trypsin-EDTA solution and ROS accumulation was measured by flow cytometry. Similarly, levels of mitochondrial ROS were detected by measuring fluorescence level of recombinant HyPer-mito sensor protein in stably expressing cells [43]. For both DCF-DA and HyPer-mito the fluorescent emission was measured using Epix XL4 flow fluorimeter (Beckman Coulter) at a wavelength of 535 nm, with excitation at a wavelength of 488 nm.

### Measurement of cell viability

Cells were seeded into a 96 well plate (5000 cells per well). After 24 hours cells were exposed to 900–1200 µM hydrogen peroxide for 20 hours. Viability was determined by adding 20 µl per well of the Cell Titer Blue reagent (Promega). The fluorescent signal was measured by the Plate Chameleon V microplate reader under 544 nm excitation and the fluorescence emission was measured at a wavelength of 590 nm.

### Mitochondrial staining and detection of mitochondrial fragmentation

Cells were seeded into 35 mm plates (80,000 cells per plate). Twenty four hours after seeding cells were exposed to varying concentrations of hydrogen peroxide and incubated for 20 hours. The cells were then stained with Mitotracker Red (Invitrogene, 100 nM) at 37°C for 15 min under 5% CO_2_. The cells were incubated further without the dye under the same conditions for 15 min. Pictures were taken by viewing with Leica DMI4000B inverted fluorescence microscope. The degree of mitochondrial fragmentation was evaluated by analyzing of 200 cells in each plate.

### Western analysis

Cells were subjected to a lysis buffer MPER (Pierce) supplemented with a protease inhibitors cocktail (Complete Mini, Roche). Protein extracts were resolved by 12% SDS-polyacrylamide gel electrophoresis. Antibodies that recognize all protein isoforms of the *SHC1* gene were used for detection (610082, BD Biosciences). GAPDH antibodies (sc32233, Santa Cruz) were used as a loading control. Quantitative assessment of protein bands was conducted by densitometry and analysis using the ImageJ program.

## Results

### Generation of human cell lines with knocked-down and overexpressed p66shc

**Figure 1:**
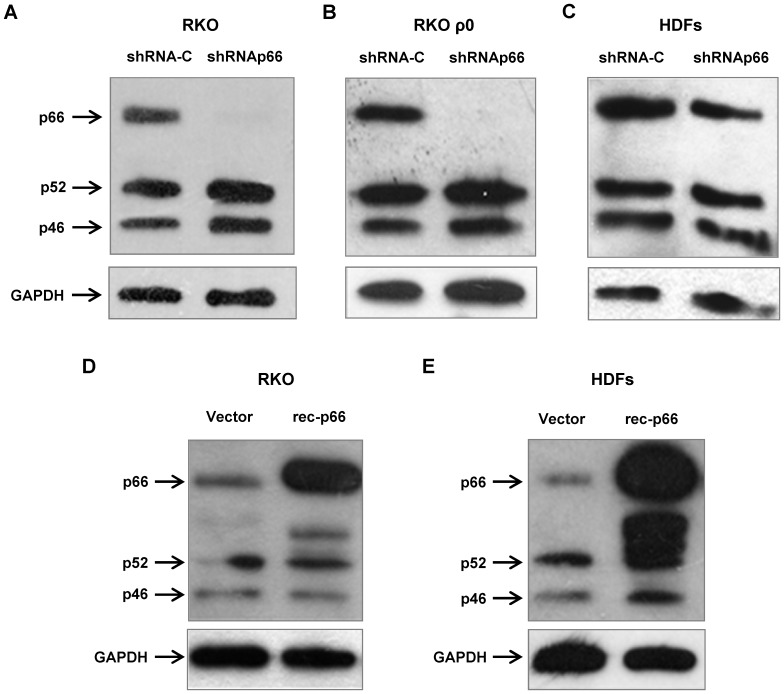
Western analysis of *SHC1* gene products in human colon carcinoma derived cell line RKO and in normal human dermal fibroblasts (HDFs). (A) RKO cells; (B) mitochondria-depleted ρ0 RKO cells; (C) HDFs. The cell lines were infected with either control lentiviral vector expressing random shRNA (shRNA-C), or with a lentiviral construct expressing shRNA against CH2 domain of p66shc (shRNAp66). (D) RKO cells and (E) HDFs, control (Vector) or with introduced lentiviral construct overexpressing recombinant p66shc (rec-p66).

To study a role of p66shc in ROS production during oxidative stress in human cells we generated sublines of human colon carcinoma cells RKO and diploid human dermal fibroblasts (HDFs). Stable knockdown of p66shc expression was achieved by an introduction of a lentiviral construct pLSLP expressing shRNA targeting a unique region in the p66shc mRNA that is absent in the p52shc and p46shc isoforms. After selection of the transduced RKO cells with puromycin there was almost complete inhibition of p66shc expression, while the expression of the p52shc and p46shc isoforms was not affected (Fig. 1A). A similar level of p66shc mRNA inhibition was observed in the ρ0 derivative of RKO cells, which does not contain mitochondrial DNA and has no functional ETC (Fig. 1B). In the transduced and purimycin-selected HDFs the expression of p52shc and p46shc isoforms were also unaffected, although the inhibition of the p66shc protein expression was less prominent (55% as compared to a control) (Fig. 1C).

For overexpression of p66shc we introduced into the cells a lentiviral construct pLCMV-p66shc in which the transcription is initiated from a strong CMV-promoter. Empty pLCMV vector was used as a control. There was a 5-fold (Fig. 1D) and 15-fold (Fig. 1E) increase in the expression of p66shc in RKO cells and in HDFs, respectively.

### Participation of p66shc in the hydrogen peroxide induced oxidative stress

We examined the effect of p66shc expression on the elevation of intracellular ROS after treatment with 1200 µM of exogenous hydrogen peroxide, which was capable to induce oxidative stress both in RKO cells and HDFs. After 3 hours there was a significant accumulation of intracellular ROS detected by by DCF-DA staining and FACS analysis. The intracellular ROS apparently have an endogenous origin since the hydrogen peroxide added to cell cultures is degraded within half an hour [44].

**Figure 2:**
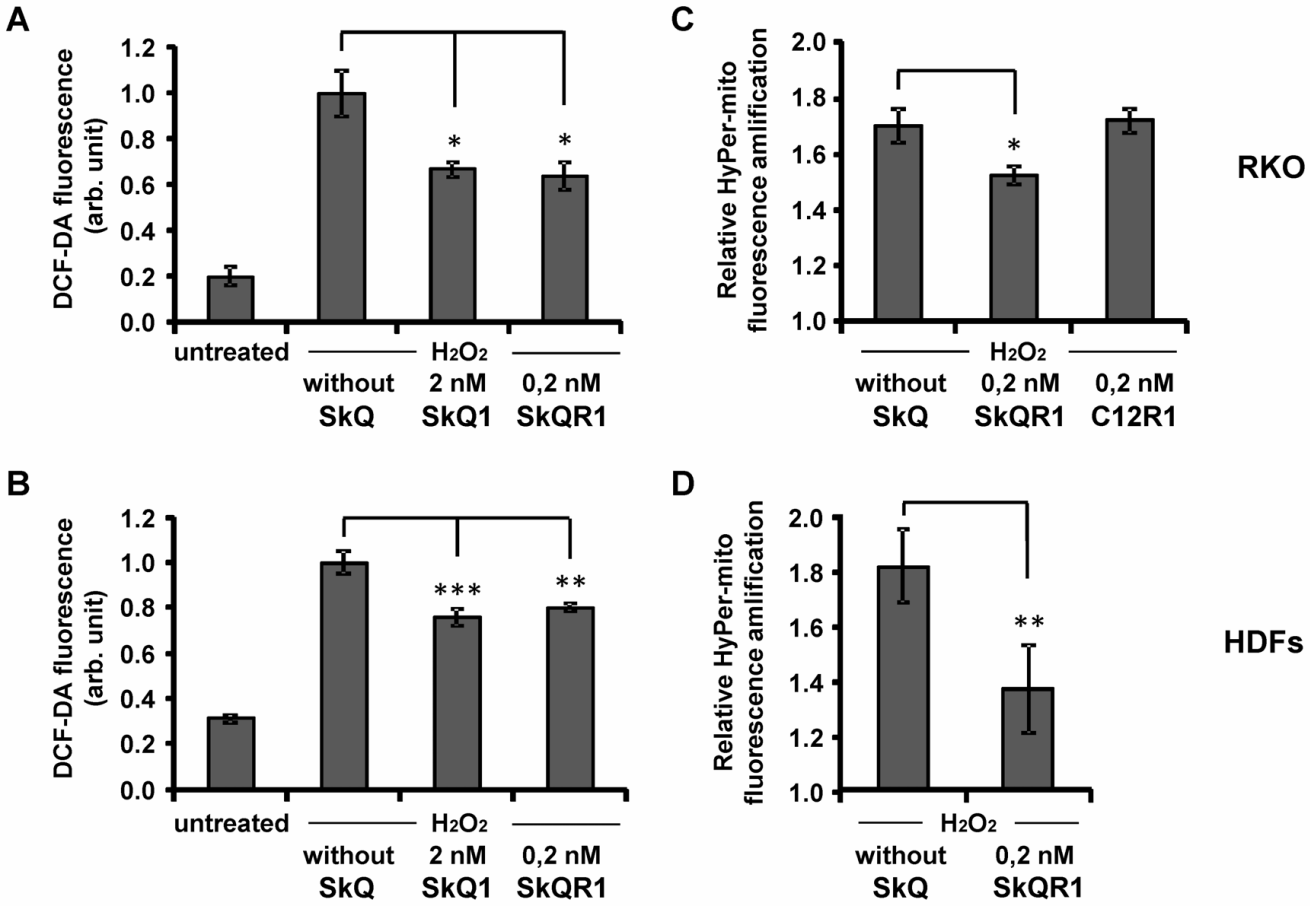
Mitochondria-targeted antioxidants decrease intracellular and mitochondrial ROS induced by hydrogen peroxide in RKO cells and in HDFs. RKO cells and HDFs were incubated with indicated concentrations of SkQ1 or SkQR1 during 3 days. Mitochondria-targeted compound C12R1 (dodecylrhodamine), a derivative of SkQR1 without plastoquinone [56] was used as a control lacking antioxidant activity. Basal and hydrogen peroxide-induced (3 hours) ROS levels in RKO cells (A) and HDFs (B) treated (or non-treated) with SkQR1, measured by fluorescence-activated cell sorting (FACS) after DCF-DA staining. Cells that express HypPer-mito were preincubated with indicated concentrations of SkQR1. Amplification of HyPer-mito fluorescence in (C) RKO cells and (D) in HDFs treated with 1200 µM hydrogen peroxide during 3 hours. The results showing DCF-DA fluorescence and amplification of HyPer-mito fluorescence are from three parallel cell cultures, *p<0.05; **p<0.01; ***p<0.001 in paired t-test. The data were replicated in three independent experiments.

Mitochondria-targeted antioxidants are useful tools for elimination of ROS produced in mitochondria [45]. Preincubation with mitochondria-targeted antioxidants SkQ1 (2 nM) and SkQR1 (0.2 nM) resulted in an essential decrease in ROS level induced by the exposure to hydrogen peroxide (Fig. 2A, B). For further evaluation of mitochondrial ROS production we introduced into RKO cells and HDFs a lentiviral construct expressing mitochondria-targeted recombinant fluorescent protein HyPer-mito, a sensor for hydrogen peroxide in mitochondria [43]. Mitochondrial localization of the protein was confirmed by fluorescent microscopy (not shown). There was an increase in the mitochondrial fluorescence 3 h after the addition of 1200 µM hydrogen peroxide for both cell lines, while the preincubation of the cells with 0.2 nM SkQR1 decreased substantially the response (Fig. 2C, D). Consistent with our previous observations [44] the data suggest that mitochondria serve as one of the major sources of ROS in the model of oxidative stress induced by hydrogen peroxide.

**Figure 3:**
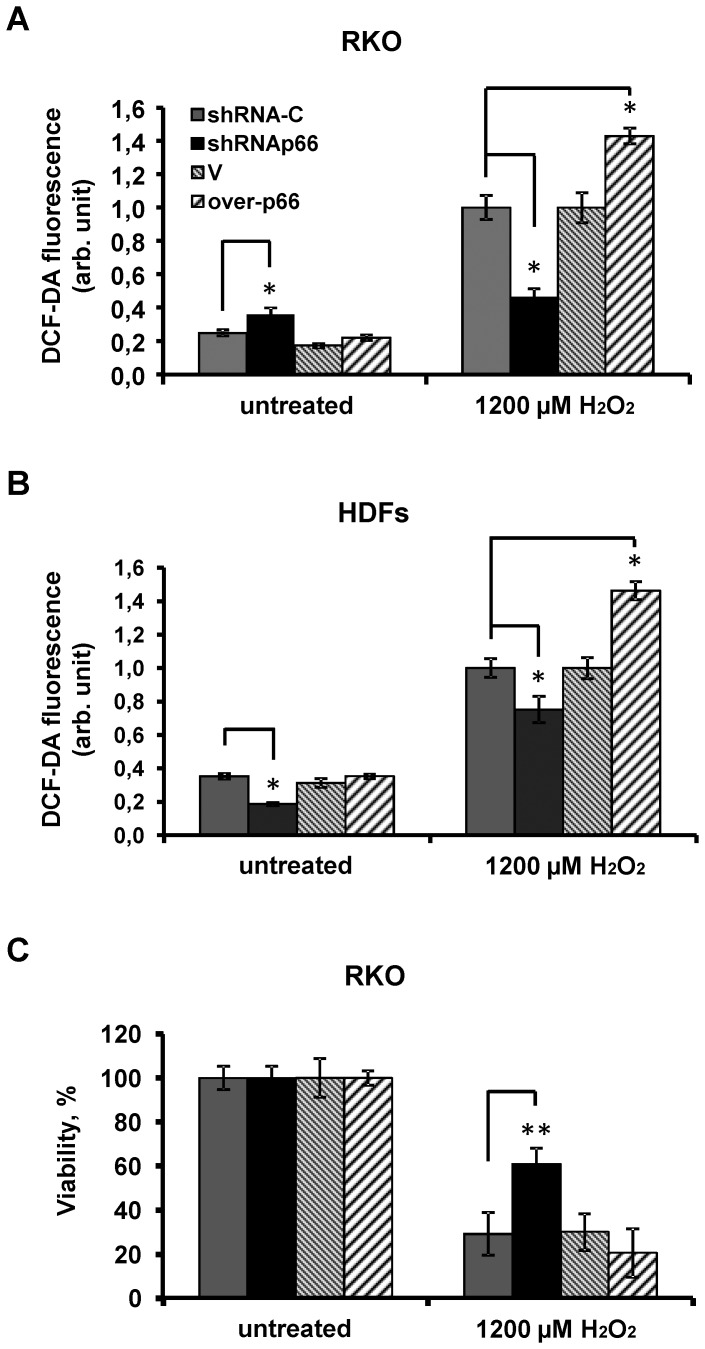
p66shc knockdown decreases but the overexpression auguments the oxidative stress induced by hydrogen peroxide in RKO cells and in HDFs. (A) DCF-DA fluorescence level in RKO cells after an exposure to hydrogen peroxide for 3 hrs at the indicated concentrations. (B) Viability of RKO cells was measured after exposure to hydrogen peroxide (1200 µmol) for 20 hrs. (C) DCF-DA fluorescence level in HDFs after exposure to hydrogen peroxide for 3 hrs at indicated concentrations. Grey columns correspond to cells transduced with control random shRNA (sh-C); black columns correspond to cells that express shRNAp66; grey diagonal hatched columns – cells expressing control empty vector and light diagonal hatched columns refer to cells that overexpress p66shc. Mean values of DCF fluorescence intensity (viability) that are measured in three (five) parallel cell cultures are represented. *p<0.05; **p<0.01 in paired t-test. Data were replicated in three independent experiments.

Knocking down the expression of p66shc in RKO cells by shRNA resulted in a 2-fold decrease, while an overexpression of p66shc has led to substantially increased levels of the stress-induced intracellular ROS (Fig. 3A). Similar effects were observed in the diploid HDFs, highlighting the pro-oxidant activities of p66shc (Fig. 3B).

The hydrogen peroxide induced oxidative stress affected negatively viability of RKO as measured 20 h after the addition of hydrogen peroxide. There was good inverse correlation between the induced levels of ROS and the number of viable cells (Fig. 3C).

**Figure 4:**
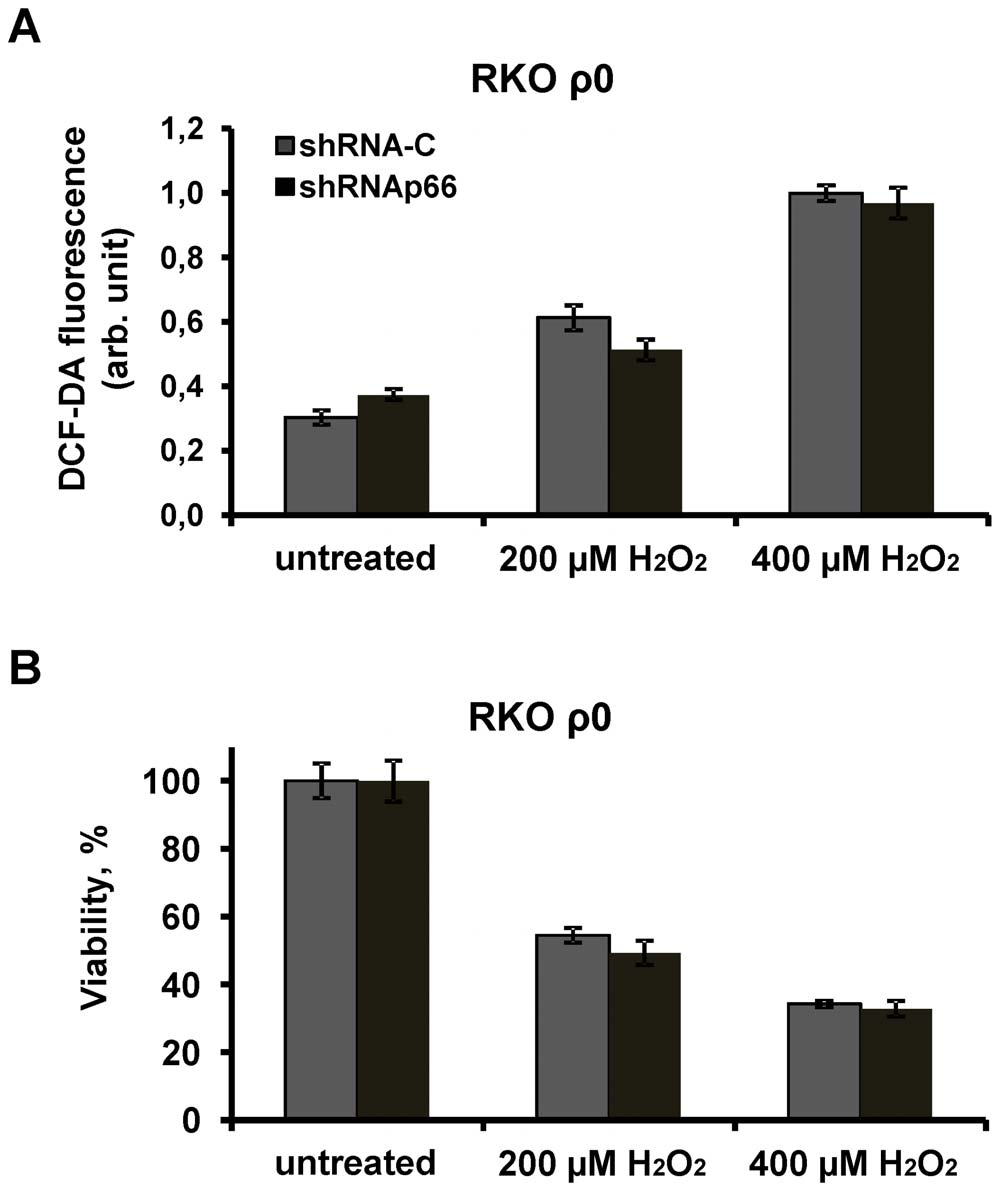
Pro-oxidant properties of p66shc are inhibited in RKO ρ0 cells. (A) Cell viability was measured after exposure to hydrogen peroxide for 20 hrs at indicated concentrations. (B) DCF-DA fluorescence level in cells after exposure to hydrogen peroxide for 3 hrs at indicated concentrations. Grey columns correspond to cells transduced with control shRNA (sh-C); black columns correspond to cells that express shRNAp66. Represented are the fluorescence intensity values (cell viability) that are measured in three (five) parallel cell cultures. Data were replicated in three independent experiments.

To further investigate the role of mitochondria in the pro-oxidant effects of p66shc we tested RKO ρ0 cells with inactive ETC that are not expected to contribute to intracellular ROS production. As the RKO ρ0 cells were found to be more sensitive to hydrogen peroxide exposure we used lower doses of the oxidant. There was a 2-fold and a 3-fold increase in intracellular ROS levels in RKO ρ0 cells 3 h after addition of 200 and 400 µM of hydrogen peroxide, respectively (Fig. 4A). Knockdown of p66shc did not affect ROS accumulation and cell viability following the treatment of RKO ρ0 cells with two doses of hydrogen peroxide (Fig. 4B). These data are consistent with the major role of mitochondria in the generation of hydrogen peroxide mediated by p66shc.

### p66shc stimulates fragmentation of mitochondrial network in response to hydrogen peroxide induced oxidative stress

**Figure 5:**
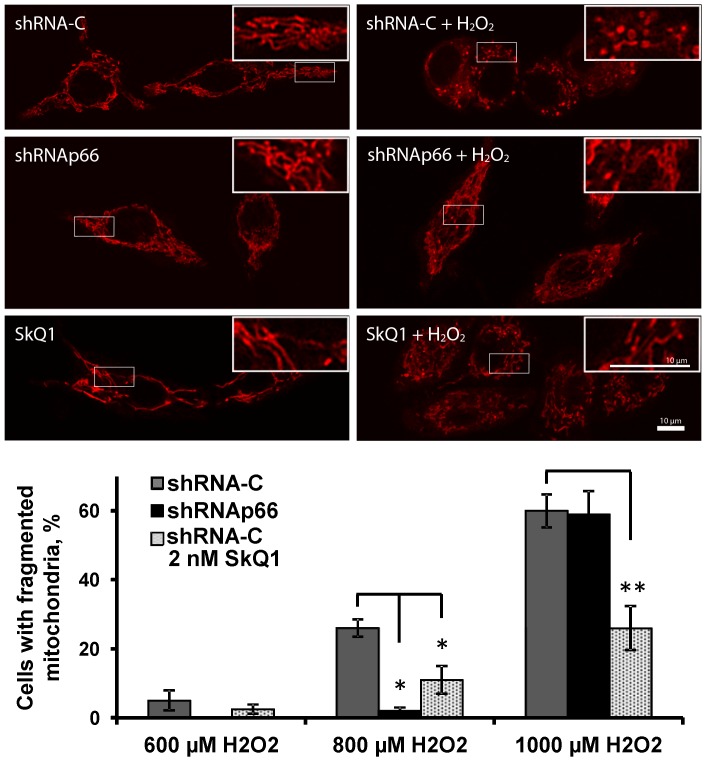
Genetic knockdown of p66shc and preincubation with SkQ1 prevents fragmentation of mitochondria induced by hydrogen peroxide in RKO cells. Fragmentation of mitochondria in different sublines of RKO cells after exposure to hydrogen peroxide for 20(sh-C); black columns correspond to cells that express shRNAp66, hatched columns – cells transduced with control shRNA and preincubated with 2 nM SkQ1 for 24 hours. Mean and standard deviation values are provided. *p<0.05; **p<0.01 in paired t-test. Data were replicated in three independent experiments.

A fragmentation of mitochondrial network represents one of the early manifestations of oxidative stress [46]. We tested the influence of p66shc on mitochondrial network fragmentation during the hydrogen peroxide induced oxidative stress in RKO cells. The inhibition of p66shc by shRNA did not cause any significant changes in morphology and distribution of mitochondria in RKO cells. Treatment of control RKO cells with a sublethal dose of hydrogen peroxide (800 µM) for 20 h induced a significant fragmentation of mitochondrial network, which was prevented by preincubation with 2 nM of mitochondrial antioxidant SkQ1. The fragmentation was virtually undetectable in the similarly treated (800 µM hydrogen peroxide) RKO cells with knocked down expression of p66shc (Fig. 5). However, the fragmentation started to emerge in the p66shc knocked down RKO cells at the hydrogen peroxide concentrations above 1000 µM, while the protective effect of SkQ1 remained well pronounced (Fig. 5).

### Participation of p66shc in the oxidative stress induced by serum deprivation

**Figure 6:**
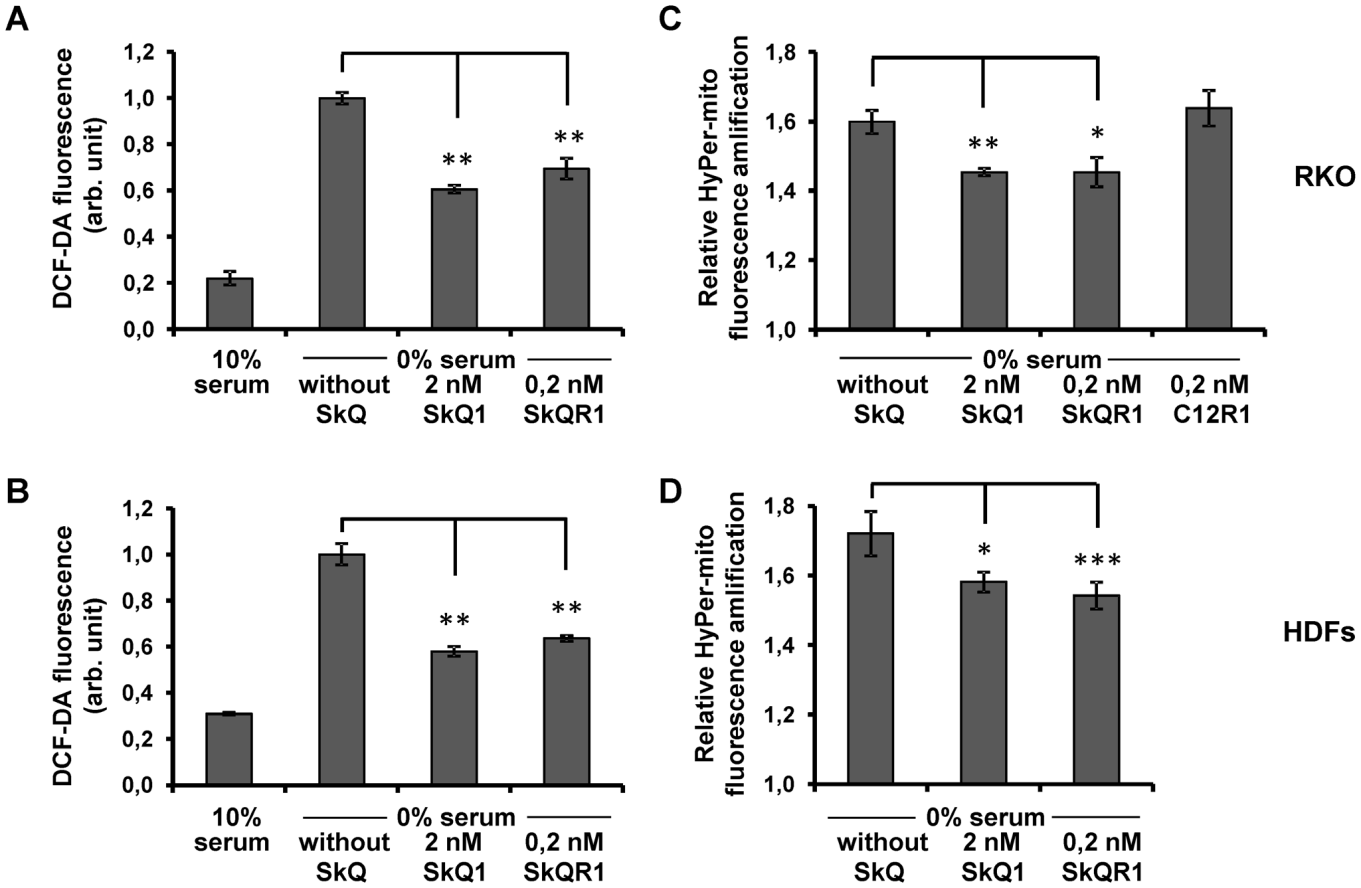
Mitochondria-targeted antioxidants decrease intracellular and mitochondrial ROS induced by serum deprivation in RKO cells and in HDFs. RKO cells and HDFs were incubated with indicated concentrations of SkQ1 or SkQR1 for 3 days. Quantification of DCF-DA fluorescence was performed in control RKO cells (A) and HDFs (B) and in cells cultivated in a serum free medium for 24 hours and pretreated with different concentrations of SkQ1 and SkQR1. Cells that express HyPer-mito were preincubated with indicated concentrations of SkQ1 or SkQR1. Amplification of HyPer-mito fluorescence was shown in (C) RKO cells, (D) HDFs after cultivation in serum-free medium during 24 hours. Represented are DCF-DA fluorescence and amplification of HyPer-mito fluorescence in three parallel cell cultures, *p<0.05; **p<0.01 in paired t-test. Data were replicated in three independent experiments.

Serum deprivation is an alternative model of an induced mitochondria-mediated oxidative stress [47], [48]. Incubation of RKO cells and HDFs in serum-free medium resulted in a 5-fold and a 3-fold increase in intracellular ROS levels, respectively (Fig. 6A,B). The ROS accumulation was efficiently prevented by a preincubation with the mitochondria-targeted antioxidants SkQ1 (2 nM) and SkQR1 (0,2 nM). To test specifically the role of mitochondria in the production of ROS we introduced into the cells a construct expressing hydrogen peroxide sensor protein HyPer-mito. The HyPer-mito fluorescence was substantially intensified in response to serum deprivation in the both cell types while the preincubation with mitochondrial antioxidants SkQ1 and SkQR1 reduced the effect (Figures 6C,D).

**Figure 7:**
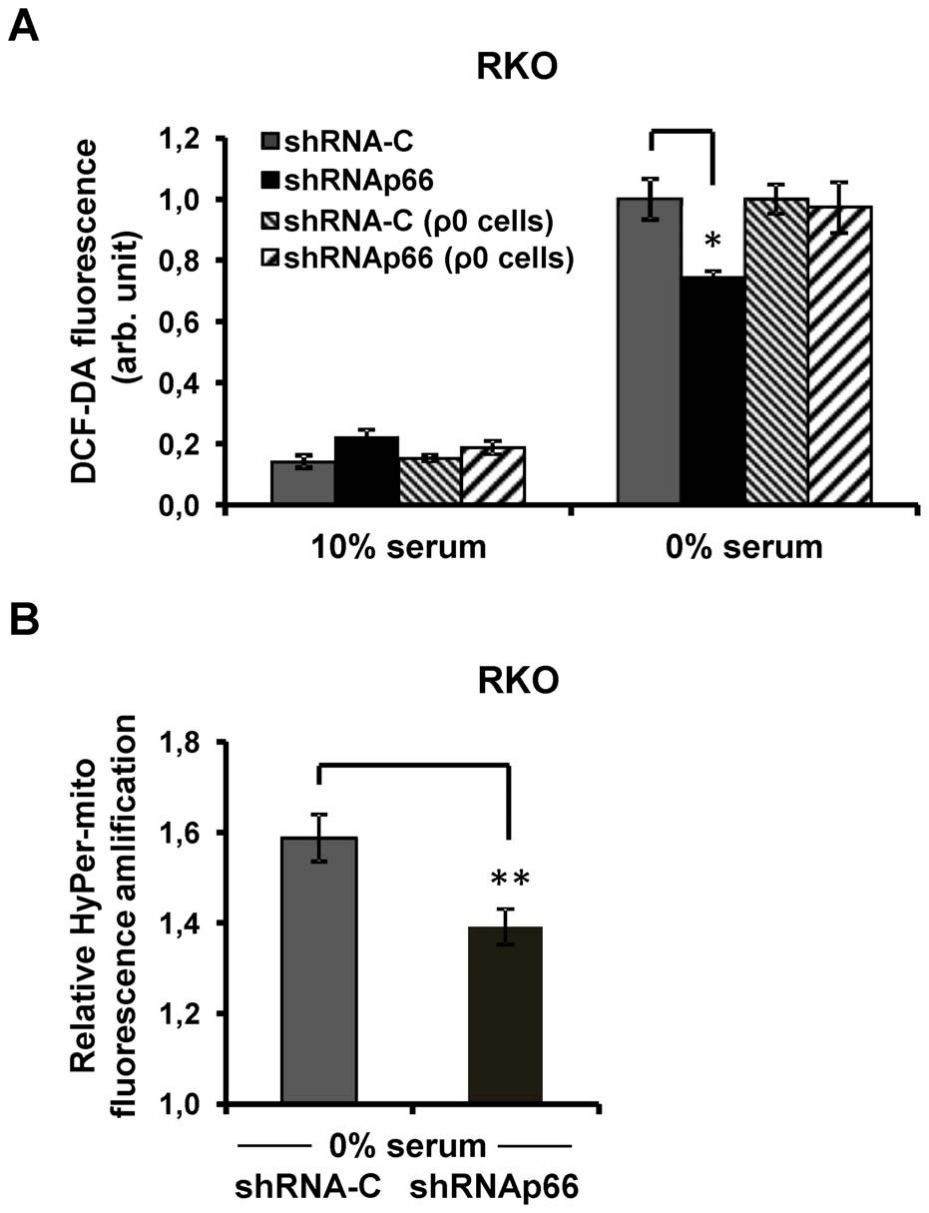
p66shc knockdown inhibits mitochondrial ROS production induced by serum deprivation in RKO cells. (A) DCF-DA fluorescence level in RKO and RKO ρ0 cells in standard media after an incubation in a serum free medium for 24 hrs. (B) Amplification of HyPer-mito fluorescence in RKO cells after an incubation in a serum-free medium for 24 hrs. Grey columns correspond to RKO cells transduced with a control shRNA (sh-C); black columns – RKO cells that express shRNAp66; grey diagonal hatched columns – RKO ρ0 cells expressing control empty vector and light diagonal hatched columns refer to RKO ρ0 cells that express shRNAp66. Represented are DCF-DA fluorescence and amplification of HyPer-mito fluorescence in three parallel cell cultures,*p<0.05; **p<0.01 in paired t-test. Data were replicated in two independent experiments.

In the RKO cells with the p66shc expression inhibited with shRNA there was a substantially less prominent elevation of both total intracellular ROS detected by DCF-DA fluorescence (Fig. 7A) and mitochondrial ROS detected with the sensor protein HyPer-mito (see Fig. 7B). In contrast, khocking down of p66shc did not have any effect on the serum deprivation-induced elevation of ROS in the ETC-deficient RKO ρ0 cells (Figure 7A).

## Discussion

To reveal an involvement of p66shc in the production of ROS during oxidative stress in human cells, and to establish the predominant source of the produced ROS we used a colon carcinoma cell line RKO and diploid human dermal fibroblasts (HDFs). Levels of p66shc were modulated by a lentivirus-mediated transfer of constructs expressing either specific shRNA targeting the p66shc mRNA region corresponding to the N-terminal domain, which is absent in p52shc and p46shc isoforms, or the p66shc mRNA governed by a strong CMV promoter. In both cell types the expression shRNA did not affect p52shc and p46shc. We have succeeded to obtain very strong suppression of p66shc (more than 90%) in RKO cells. However a much weaker inhibition (∼50%) was achieved in HDFs, possibly due to a less efficient transcription of shRNA in these cells. Therefore, while the knockdown phenotypes observed for the RKO cells could approximate to those of a gene knockout, the phenotypes of the corresponding HDFs subline are apparently partial.

As a model of oxidative stress we used a treatment with hydrogen peroxide that induces an overproduction of mitochondrial ROS [49]. The mechanism of oxidative stress following the treatment represents an example of a more general phenomenon known as a ROS-induced ROS release (RIRR) [50], which involves an induction of mitochondrial permeability transition. RIRR can initiate a chain reaction of channel opening and ROS release by neighboring mitochondria eventually leading to apoptosis. Through the mechanism the apoptotic signal can be spread for long distances from cell to cell [51]. The mechanism participates in the development of various pathophysiology manifestations of oxidative stress and, as well as in the process of a distant cell-to-cell transduction of apoptotic signals underlying certain bystander effects [52].

As a second model of oxidative stress induction we used serum deprivation, which is also associated with a release of endogenous ROS [48]. Incubation of many cell types in the medium without serum and growth factors induces apoptosis associated with a mitochondrial permeability transition, loss of mitochondrial membrane potential [53] and a production of hydrogen peroxide from the released superoxide anions [54], [55]. Mitochondria were specifically identified as being source of ROS induced by serum deprivation [47].

For an evaluation of a mitochondrial origin of ROS in these models of oxidative stress we used three approaches that included (i) administration of mitochondria-targeted antioxidants SkQ1 and SkQR1 [56], [57], (ii) introduction into cells of HyPer-mito, a mitochondria-targeted fluorescent protein sensor for hydrogen peroxide [43] and (iii) analysis of mitochondrial DNA-depleted ρ0 cells lacking the electron transport chain [38]. We have shown that 3 h after treatment with hydrogen peroxide there was a 3–5-fold increase in cytosolic ROS and almost a 2-fold increase in mitochondrial ROS detected with HyPer-mito, leading to a 3-fold loss of viability by 20 h. The mitochondria-targeted antioxidants partially prevented the loss of viability and the accumulation of cytosolic and mitochondrial ROS. The effects were similar in both RKO cells and in HDFs. The data suggest a substantial stimulation of mitochondrial ROS production following treatment with hydrogen peroxide thus contributing to the development of oxidative stress.

As another model for studying the role of mitochondria in p66shc-dependent oxidative stress we used RKO ρ0 cells devoid of mitochondrial DNA and thus lacking functional ETC. These cells were more sensitive to hydrogen peroxide than normal RKO cells probably because RKO ρ0 cells have an increased content of Fe and other metal ions that participate in generation of ROS in the Fenton reaction [58]. Knockdown of p66shc affected neither the endogenous ROS accumulation, nor the cell viability confirming that the pro-oxidant effect of p66shc is associated with mitochondria.

Knockdown of p66shc alleviates the hydrogen peroxide-induced accumulation of intracellular ROS while overexpression of p66shc results in an increase of ROS by more than 40% in both cell types. Oxidative stress induced by the hydrogen peroxide resulted in death of 70% of cells 20 h after the treatment, while the knockdown of p66shc improved substantially the viability. The result is similar to the reported effect of p66shc knockout in MEFs that almost completely prevented the hydrogen peroxide-induced cell death [18]. Partial protection against the hydrogen peroxide-induced cell death following knockdown of p66shc was also reported for human retinal pigmented epithelial cells [31]. The observed variations in the magnitude of the protective effect most likely reflect cell-type specific responses to hydrogen peroxide and different experimental conditions used in the studies.

For further clarification of the role of p66shc in oxidative stress we studied mitochondrial fragmentation, which as we have shown earlier depends on ROS accumulation inside the mitochondria [59]. The conclusion was supported by the observation that protection against the hydrogen peroxide induced mitochondrial fragmentation was the early effect of SkQ1 and its analogs, while mitigation of ROS accumulation in cytosol and death of HeLa cells or human diploid fibroblasts were observed later [59]. We observed a similar dynamics in RKO cells: while the protection against cytosolic ROS accumulation and cell death required a pre-incubation with SkQ1 for as long as 3 days, a prevention of the mitochondrial fragmentation took only 6 hours (data not shown). Knockdown of p66shc was also able to partially prevent fragmentation of mitochondria at 20 h, after addition of a sub-lethal dose of hydrogen peroxide. The result is consistent with the data published for mouse embryo fibroblasts where the p66shc knockout was protective against mitochondrial fragmentation observed in the control MEFs 30 min after the addition of hydrogen peroxide [60]. Noteworthy, at higher doses of hydrogen peroxide knockdown of p66shc was no longer protective against mitochondrial fragmentation while the effect of SkQ1 remained well pronounced (Fig. 5) indicating that the p66shc-mediated mechanism does not serve as an exclusive source of ROS production in mitochondria in this model of oxidative stress.

Serum deprivation induces the mitochondria-mediated apoptosis that includes the mitochondrial permeability transition, loss of mitochondrial membrane potential [53] as well as a production of hydrogen peroxide from the released superoxide anions [54], [55], [61]. Mitochondria were specifically identified as being source of ROS induced by serum deprivation [47]. Inhibitors of complex III myxothiazol and stigmatellin are able to decrease the ROS level induced by serum deprivation [48] indicating that complex III might contribute to ROS generation during stresses. Antioxidant treatment inhibits the apoptotic cell death, although the cells fail to proliferate in the media deprived from growth factors [62]. Romo1, a short (79 amino acids) protein localized in mitochondria [63] was recently identified as a modulator of mitochondrial ROS release to cytosol [64]. In particular, Romo1 is required for the oxidative stress induced by serum deprivation [48]. The release of mitochondrial ROS to cytosol depends on permeability of mitochondrial membrane as overexpression of Bcl-XL efficiently prevents both the Romo1-dependent ROS release and the serum-deprivation induced apoptosis [65]. As both p66shc and Romo1 participate in cytosolic release of mitochondrial ROS it would be important to test their possible functional cooperation during the induction of oxidative stress.

Although the results indicate that p66shc participates in mitochondrial ROS production in human normal and cancer cells, the exact mechanism of the function is still obscure. It was previously suggested that in mitochondria p66shc may act as an oxidoreductase that produces ROS by transferring electrons from cytochrome *c* to molecular oxygen [22]. However p66shc does not contain any known redox-active or metal-binding domains. Another explanation could be based on the known ability of cytochrome *c* to generate ROS upon its oligomerization, as a result of autooxidation [66], [67]. As p66shc can bind to cytochrome *c*, it could potentially assist in the oligomerization and autoxidation, similarly to the function of prothymosine α [68]. More recently few additional explanations have been suggested. It was found that p66shc -/- MEFs display a reduced mitochondrial oxidative phosphorylation, whereas the reliance on glycolysis is increased. [69]. In line with the observations it was found that p66shc may function as a scaffold for a signallosome complex bringing together PKCδ and cytochrome *c*, while retinol could serve as an electron carrier from C1b zinc-finger domain of PKCδ to oxidised cytochrome *c*. It was suggested that the PKCδ signalosome can form a feedback loop that measures the workload of the respiratory chain by sensing the redox balance between the oxidized and reduced cytochrome *c* and conveys signals to pyruvate dehydrogenase complex to adjust the TCA cycle influx [70]. As mitochondrial electron flow is the major producer of cellular ROS, the results may explain why p66shc deficient cells and animals have lower levels of ROS.

Noteworthy, most studies challenging functions of p66shc in mitochondria were performed in mouse models. However, the impact of oxidative stress that hypothetically may serve as a rheostat for the lifespan regulation differs substantially in the mouse and the human models. Human cells are more resistant to oxidative stresses [71] and have at least two-fold lower mitochondria ROS production rate [72]. Therefore, some mechanisms related to p66shc and their significance could also differ in human cells. More focused studies on p66shc mechanisms in human cells would provide valuable clues for treatment and prevention of accelerated aging and numerous diseases associated with elevated ROS.
